# MA104 cell line is permissive for human bocavirus 1 infection

**DOI:** 10.1128/jvi.01539-24

**Published:** 2025-01-23

**Authors:** Jielin Tang, Sijie Chen, Yijun Deng, Junjun Liu, Dan Huang, Muqing Fu, Bao Xue, Canyu Liu, Chunchen Wu, Fan Wang, Yuan Zhou, Qi Yang, Xinwen Chen

**Affiliations:** 1 Guangzhou National Laboratory 612039 , Guangzhou, China; 2 Guangzhou Medical University 26468 , Guangzhou, China; 3 GMU-GIBH Joint School of Life Sciences, Guangzhou Medical University 26468 , Guangzhou, China; 4 State Key Laboratory of Virology, Wuhan Institute of Virology, Center for Biosafety Mega-Science, Chinese Academy of Sciences 74614 , Wuhan, China; 5 Guangzhou Institutes of Biomedicine and Health, Chinese Academy of Sciences 74627 , Guangzhou, China; 6 Department of Laboratory Medicine, Maternal and Child Health Hospital of Hubei Province, Tongji Medical College, Huazhong University of Science and Technology 12403 , Wuhan, China; Cornell University Baker Institute for Animal Health, Ithaca, New York, USA

**Keywords:** human bocavirus 1, MA104, monkey kidney epithelial cell line, susceptibility, the complete life cycle

## Abstract

Human bocavirus 1 (HBoV1) has appeared as an emerging pathogen, causing mild to life-threatening respiratory tract infections, acute otitis media, and encephalitis in young children and immunocompromised individuals. The lack of cell lines suitable for culturing replicative viruses hinders research on HBoV1. Here, we characterized the susceptibility to HBoV1 of 29 human and 7 animal cell lines, and identified a permissive cell line, MA104. The complete HBoV1 life cycle was achieved in MA104 cells, including viral entry, complete replication, and infectious progeny virion production. Additionally, the suppression of the interferon pathway facilitated the viral genome replication in MA104 cells. RNA-sequencing showed that innate immunity, inflammation, the PI3K-Akt and MAPK signaling pathways, and the cellular membrane system were mobilized in response to HBoV1 infection. Overall, our study is the first to identify a cell line, MA104, that supports the complete HBoV1 life cycle, which will promote research on HBoV1 virology and pathogenesis and benefit drug and vaccine development.

**IMPORTANCE:**

HBoV1 is an emerging pathogen that mainly causes respiratory tract infections, while the lack of cell lines suitable for culture replicative viruses hindered research on HBoV1. Here, we identify a permissive cell line for HBoV1 infection, MA104, and reveal that the complete life cycle of HBoV1 was supported in MA104 cells. Our findings provide a suitable cell model for the study of HBoV1 and explore its application for antiviral drug evaluation, which is vital for research on HBoV1 virology and pathogenesis, as well as for drug and vaccine development.

## INTRODUCTION

Human bocavirus 1 (HBoV1) is a common respiratory pathogen that causes mild to life-threatening acute respiratory tract infections in children and immunocompromised individuals, with an increasing number of cases reported worldwide (1–3). Children infected with HBoV1 are more likely to suffer from various ear and respiratory illnesses, including acute otitis media, pneumonia, bronchiolitis, and asthmatic exacerbations (4, 5). The absence of cell lines for replicative virus culture and the lack of experimental animal models for HBoV1 infection seriously slow down research on the pathogenic mechanisms and antiviral drug and vaccine development.

HBoV1 belongs to the Bocaparvovirus genus of the *Parvoviridae* family. It is a small, nonenveloped, typical icosahedral virus of ~26 nm in diameter that contains a single-stranded DNA (ssDNA) genome of about 5.5 kb with hairpins at both ends (6, 7). The HBoV1 genome comprises three open-reading frames (ORF1, ORF2, and ORF3) that express three structural proteins (VP1, VP2, and VP3) and six non-structural proteins (NS1, NS1-70, NS2, NS3, NS4, and NP1) (1, 8, 9). VP1, VP2, and VP3 constitute the viral capsid in a ratio of 1:1:10 (2). The viral capsid surface carries host determinants and is involved in many processes, including host tropism, cell recognition, pathogenicity, assembly, and immune response (10, 11). NS1 and NP1, which appear to be substantially conserved, are crucial for viral DNA replication and are employed as targets for HBoV1 detection (9, 12).

Parvoviruses enter cells by receptor-mediated endocytosis, which includes the binding of virions to specific receptors on the cell surface, followed by internalization of the virions into the host cell (9, 13). However, the HBoV1 receptors remain unknown. HBoV has been detected in several tissues, including paranasal sinus mucosal (in the nasal cavity), hypertrophic adenoids (in the upper airway), lymphatic, duodenal, colorectal, and placental tissues, with HBoV1 more frequently infecting the respiratory system and gastrointestinal tract (14–21). HBoV1 only infects well-differentiated or polarized human airway epithelial (HAE) cells *in vitro*; air–liquid interface (ALI) cultures (including HAE-ALI and T84-ALI) enable HBoV1 isolation, which requires extensive and expensive cell culturing efforts (9, 22–24). While HEK293T or HEK293 cells cannot be infected with HBoV1, they can replicate the viral duplex genome, producing high titers of progeny virions upon transfection (HBoV1 cannot enter HEK293T or HEK293 cells, but they can be transfected) (22). Caco‐2 cells allow HBoV1 infection and genome replication, but infectious progeny virions are not produced (25). Cultivation of the virus in cell lines remains a major challenge.

Here, we identified a HBoV1-permissive monkey kidney epithelial cell line (MA104) and demonstrated that MA104 cells support HBoV1 entry, genome transcription and replication, and production of infectious progeny virions. Suppression of interferon (IFN) signaling significantly enhanced the viral genome replication. Altogether, the results confirm that MA104 cells support HBoV1 infection, providing a viable cell model for studies on HBoV1 infection.

## RESULTS

### MA104 cell line is a permissive cell line for HBoV1 infection

**Fig 1:**
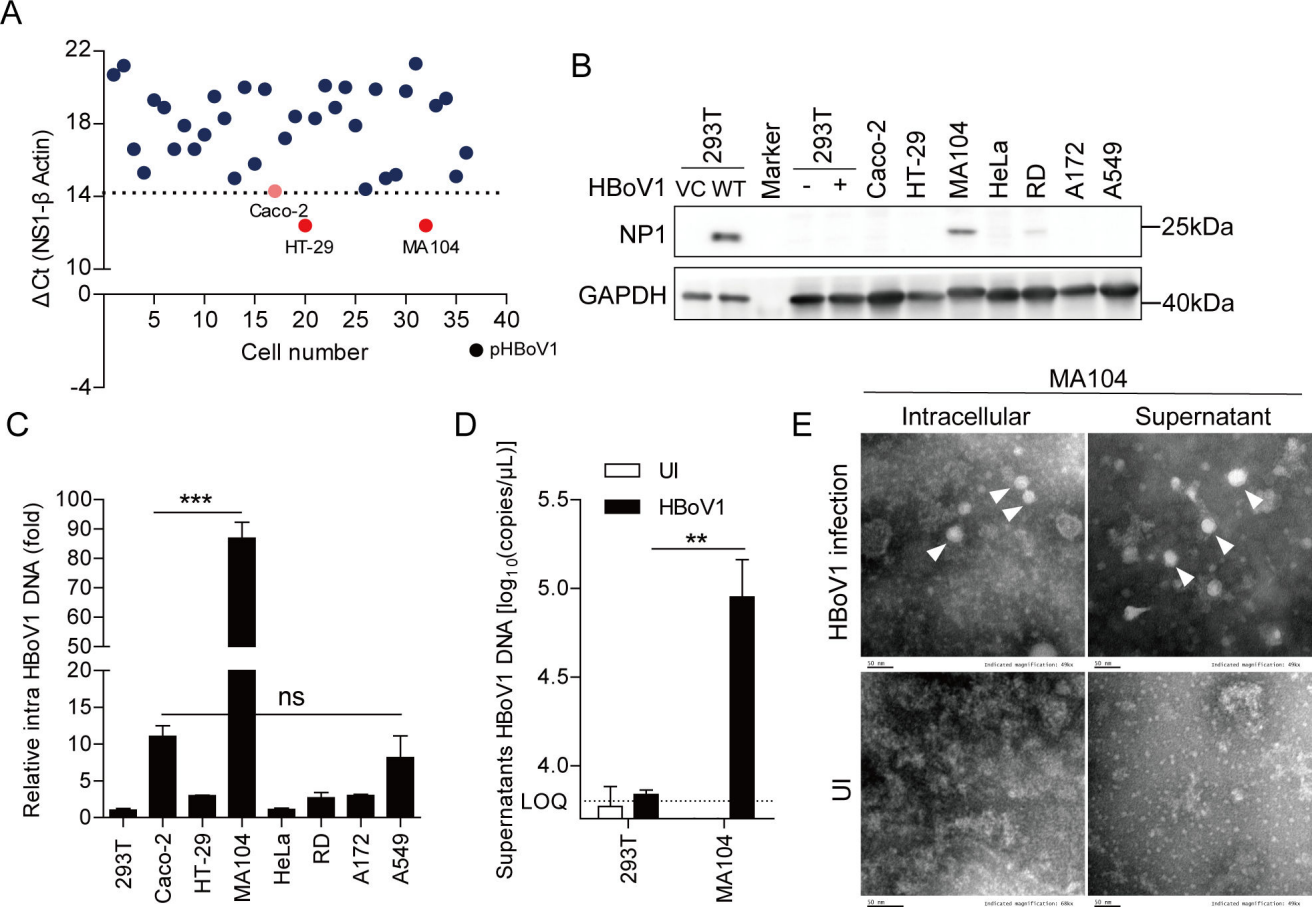
MA104 cell line is a permissive cell line for HBoV1 infection. (**A**) Cells were incubated with HBoV1 (3,000 viral genome equivalents (vge)/cell) for 12 h, and then NS1 mRNA was quantified by qRT-PCR at 72 h.p.i. (HEK293T cells transfected with pHBoV1-WH served as the positive control). (**B and C**) Cells were incubated with HBoV1 (3,000 vge/cell) for 12 h (HEK293T cells transfected with pHBoV1-WH served as the positive control), and then (**B**) NP1 protein was analyzed by Western blotting and (**C**) intracellular viral DNA copies were quantified by qPCR at 96 h.p.i. Graphs show mean ± SD; n = 3. ***P < 0.001; ns, no significant difference (one-way ANOVA in relation to Caco-2 group). (**D**) HEK293T or MA104 cells were incubated with HBoV1 (3,000 vge/cell) for 12 h, and then extracellular viral DNA copies were quantified at 96 h.p.i. Graphs show mean ± SD; n = 3. **P < 0.01 (student’s *t*-test). (**E**) MA104 cells were infected or not with HBoV1 (3,000 vge/cell) for 12 h, and then intracellular and extracellular HBoV1 virions were subjected to negative-stain electron microscopy at 96 h.p.i. Scale bars, 50 nm. Data are representative of three independent experiments.

To obtain a cell line supportive of HBoV1 infection, 29 human cell lines (derived from kidney, lung, brain, lymphocyte, intestine, muscle, thyroid, and breast tissues) and 7 animal cell lines (derived from mouse, monkey, dog, pig, and cow tissues) were evaluated (Table S1). HBoV1 NS1 mRNA was quantified by qRT-PCR to determine whether the cells were infected by the virus at 72 h post infection (h.p.i). Of the 36 cell lines, 16 were not infected (NS1 mRNA below the limit of detection). NS1 mRNA was positive in Caco-2 (colon epithelial) cells (Table S1), which is consistent with a previous report showing that Caco-2 cells support HBoV1 entry (25). Compared to the Caco-2 cells, NS1 mRNA was higher only in HT-29 and MA104 cells (Fig. 1A), indicating that these two cell lines may be positive for HBoV1 infection. NS1 mRNA was lower (Ct value＞Caco-2 cells’ value) in all other cell lines (Table S1).

Next, the viral non-structural NP1 protein in HEK293T, Caco-2, HT-29, MA104, HeLa, RD, A172, and A549 cells was detected by Western blotting. NP1 protein was detected in HBoV1-infected MA104 and RD cells, but not in other tested cells (Caco-2, HT-29, HeLa, A172, or A549 cells) or HEK293T cells (negative control, as HBoV1 cannot enter HEK293T cells) (Fig. 1B). Thereafter, the intracellular viral DNA copies were quantified by qPCR. MA104 cells, but no other cells, had more intracellular viral DNA copies than Caco-2 cells (Fig. 1C). These results provide a preliminary suggestion that MA104 cells, a monkey kidney epithelial cell line (Fig. S1), might support HBoV1 infection, which was the focus of subsequent experiments.

The increase in extracellular viral DNA copies in HBoV1-infected MA104 cell culture supernatant, based on qPCR, further indicated that MA104 cells support HBoV1 infection (Fig. 1D). Additionally, the intracellular and extracellular virions (spherical ~26-nm-diameter structures) were purified from HBoV1-infected MA104 cell lysate and cell culture supernatant, respectively, further confirming HBoV1 infection in MA104 cells (Fig. 1E). These results collectively suggest that the MA104 cell line can support HBoV1 infection.

### HBoV1 infection characterization in MA104 cells

**Fig 2:**
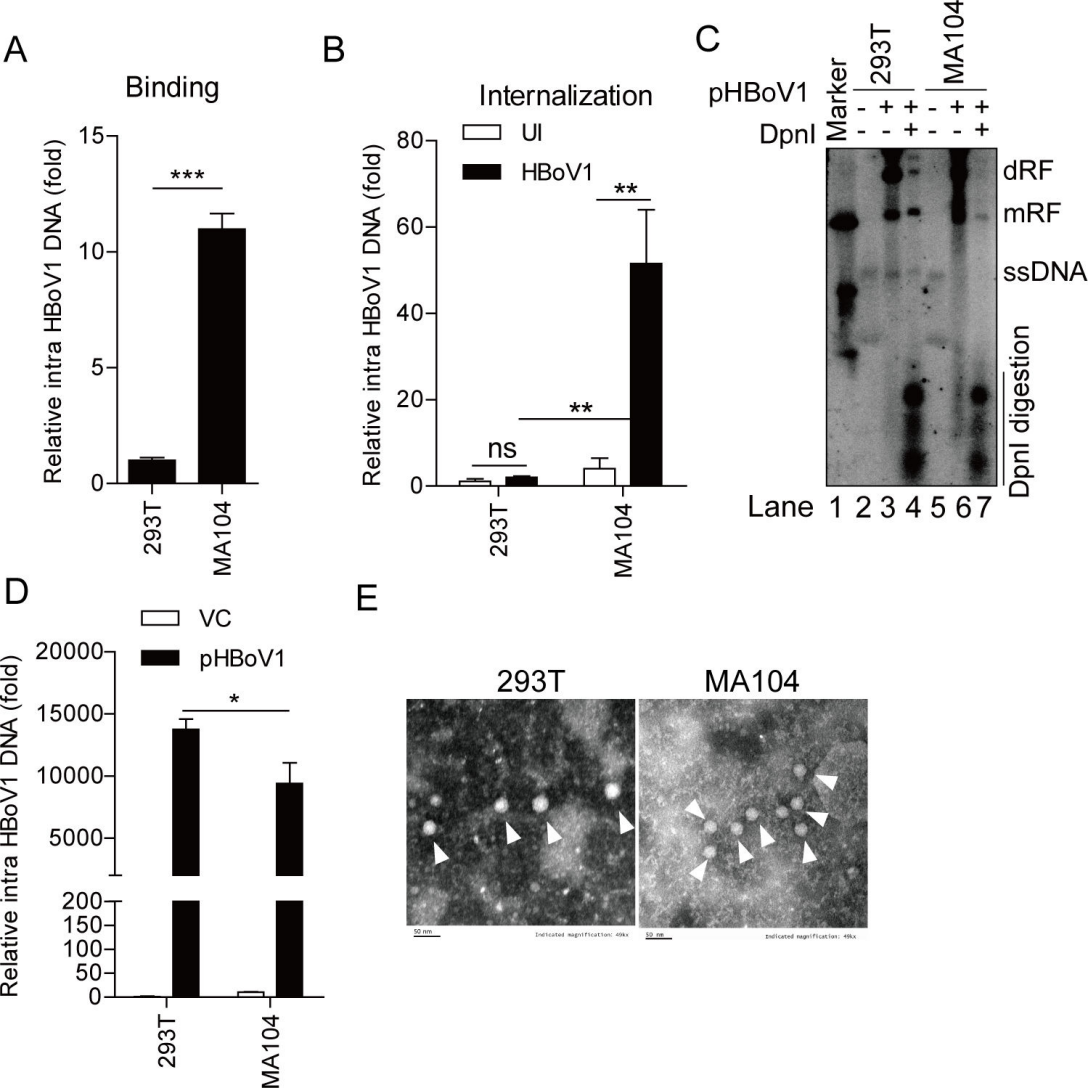
HBoV1 infection characterization in MA104 cells. (**A**) Virus binding assay. HEK293T or MA104 cells were incubated with HBoV1 at 4°C for 1 h and washed five times, and then intracellular viral DNA copies were detected by qPCR. (**B**) Virus internalization assay. HEK293T or MA104 cells were incubated with HBoV1 at 4°C for 1 h and then at 37°C for 6 h, digested using protein K (500 ng/mL; 4°C, 1 h), and washed, and then intracellular viral DNA copies were detected by qPCR. (**C**) HEK293T or MA104 cells were transfected with pHBoV1-WH or empty vector control (VC), and Hirt-extracted DNAs were digested using DpnI restriction endonuclease for 12 h and separated by 1.2% agarose gel electrophoresis. dRF (double replicative form DNA) and mRF (monomer replicative form DNA), which were resistant to digestions by DpnI restriction endonuclease, were detected in the transfected cells by Southern blotting. (**D**) HEK293T or MA014 cells were transfected with pHBoV1-WH or VC for 48 h, and then intracellular viral DNA copies were digested using DpnI restriction endonuclease and detected by qPCR. (**E**) HEK293T or MA104 cells were transfected with pHBoV1-WH for 48 h. The virions in the extracellular of MA104 cells or in the intracellular of HEK293T were purified, and then, the HBoV1 virions were subjected to negative-stain electron microscopy. Scale bars, 50 nm. Data are representative of three independent experiments. Graphs show mean ± SD; *n =* 3. *P < 0.05; **P < 0.01; ***P < 0.001; ns, no significant difference (student’s *t*-test).

The infection characterization of HBoV1 in MA104 cells was further evaluated. HBoV1 virion binding and internalization assays showed that HBoV1 could bind to MA104 cells, and HBoV1 could enter MA104 cells but not HEK293T cells (Fig. 2A and B). Then, MA104 cells were transfected with infectious clone pHBoV1-WH (26) to evaluate the HBoV1 genome replication in MA104 cells. After 48 h post-transfection, the cells were harvested and Hirt DNA was extracted for Southern blotting. Newly synthesized DNAs, i.e., DpnI digestion-resistant double replicative form (dRF) and monomer replicative form (mRF) (Lane 7) were detected in the transfected MA104 cells (Fig. 2C). Additionally, the HBoV1 replication was further confirmed by quantification of (1) intracellular viral DNA copies (after digestion using DpnI restriction endonuclease) by qPCR (Fig. 2D), (2) NP1 protein by Western blotting (Fig. S2A), and (3) NS1 protein by immunofluorescence (Fig. S2B). Furthermore, negative-stain electron microscopy revealed that purified extracellular virions (spherical ~26-nm-diameter structure) from MA104 cells were present, indicating successful virion assembly and release by MA104 cells (Fig. 2E). We also found that the replication of HBoV1 did not result in lysis or death of MA104 cell during pHBoV1-WH transfection (Fig. S2C, left) or HBoV1 infection (Fig. S2C, right). Collectively, these results suggested that HBoV1 can be internalized, replicated, assembled, and released by MA104 cells.

### Replication of HBoV1 in MA104 cells

**Fig 3:**
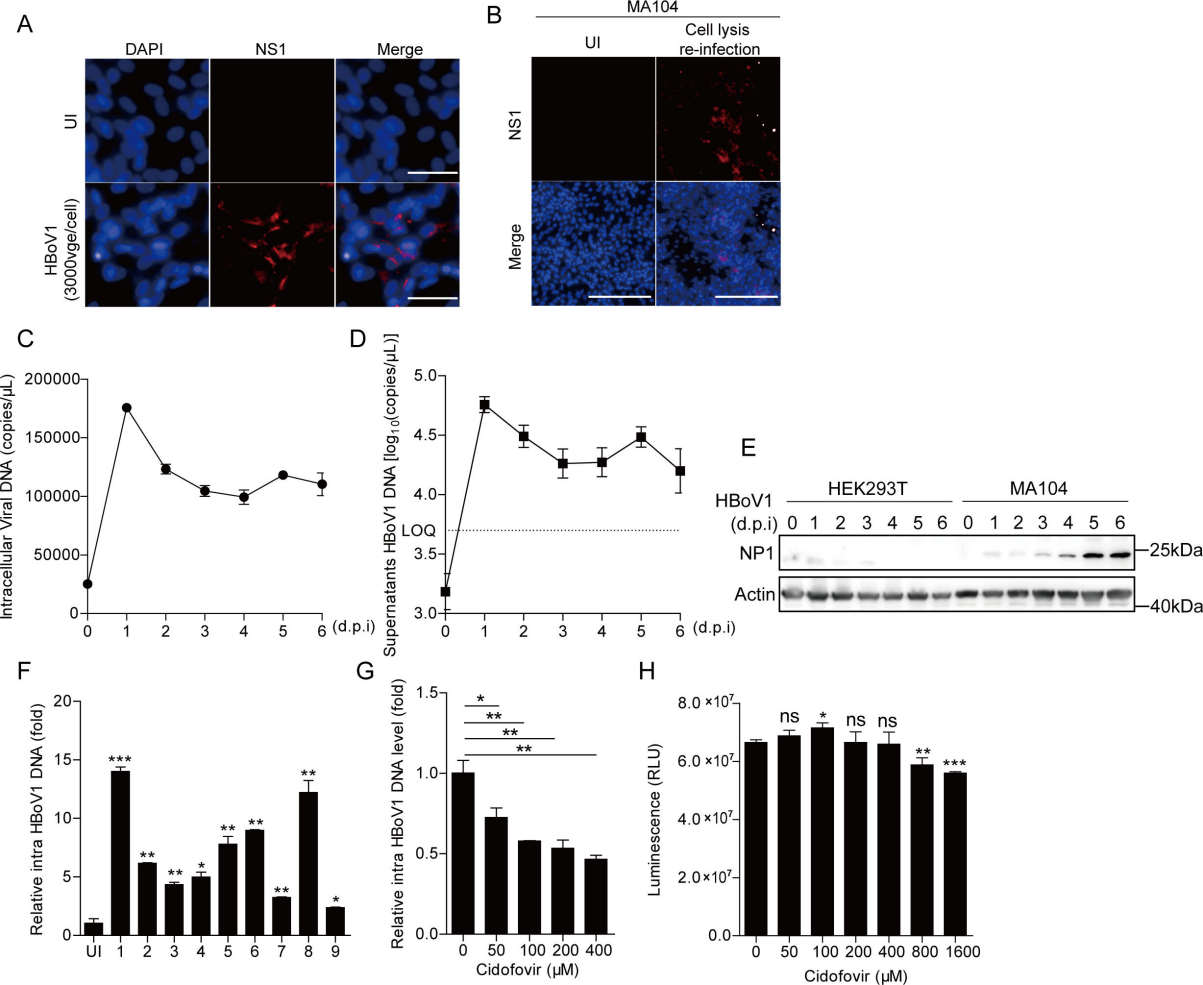
Replication of HBoV1 in MA104 cells. (**A**) MA104 cells were infected with 3,000 vge/cell of HBoV1. After 96 h.p.i., the virions infectivity was detected by fluorescent focus assay, probed with the DNA-binding dye DAPI (blue), anti-NS1 (red). Fluorescence microscopy images involve 40× magnification. Scale bar, 50 µm. (**B**) MA104 cells were incubated with supernatants of the HBoV1-infected intracellular lysis in MA104 cells. After 96 h.p.i., the progeny virions infectivity was detected by fluorescent focus assay, probed with the DNA-binding dye DAPI (blue), anti-NS1 (red). Fluorescence microscopy images involve 10× magnification. Scale bar, 100 µm. (**C and D**) MA104 cells were incubated with HBoV1 (3,000 vge/cell), and then (**C**) intracellular and (**D**) extracellular viral DNA copies were quantified at the indicated time points. (**E**) HEK293T or MA104 cells were incubated with HBoV1 (3,000 vge/cell) for 12 h, and then NP1 protein was analyzed at the indicated time points. (**F**) MA104 cells were infected using throat swabs (400 µL) from 9 HBoV-infected patients for 24 h, and then intracellular viral DNA copies were detected at 96 h.p.i. (*n* = 2) (compared with uninfected cells), *P < 0.05; **P < 0.01; ***P < 0.001 (Student'*s t*-test). (**G**) Antiviral activity of cidofovir against HBoV1. MA104 cells were treated with 50, 100, 200, 400 µM cidofovir for 6 h and then infected with HBoV1 for 12 h, and the intracellular viral DNA copies were detected at 96 h.p.i. (**H**) Cell viability was evaluated with the treatment of Cidofovir (50–1,600 µM) in MA104 cells (Compared with 0 µM control group). Graphs show mean ± SD; *n =* 3. *P < 0.05; **P < 0.01; ***P < 0.001; ns, no significant difference (Student'*s t*-test).

We further conducted an immunofluorescence-based focus-forming assay (FFA) to verify all stages of the HBoV1 life cycle in MA104 cells. Following the infection of an MA104 cell monolayer with HBoV1 (3,000 vge/cell), the virus-infected foci were typically stained with NS1-specific antibodies (Fig. 3A). The titer of the virus used for infection, measured through FFA with gradient dilution (Fig. S2D), was ~1.5 × 10^5^ FFU/mL. Additionally, progeny virions produced in the intracellular or supernatants (100 × concentration) of HBoV1-infected MA104 cells were capable of reinfecting MA104 cells, as evidenced by the detection of NS1 protein expression by immunofluorescence (Fig. 3B), and extracellular viral genome ((8.6 ± 0.07)×10^7^ copies/mL) (Fig. S2E) and NP1 protein expression (Fig. S2F). Hirt DNAs extracted from HBoV1-infected cells showed that there was a ~5 kb gel electrophoresis band successfully detected in MA104 cells, which aligned with the coding region sequence of the HBoV1 genome after second-generation sequencing (Fig. S2G). The HBoV1 replication dynamics were then monitored for 6 days post-infection (d.p.i.) with 3,000 vge/cell. QPCR analysis of HBoV1 genomes in MA104 cells revealed that intracellular (Fig. 3C) and extracellular (Fig. 3D) viral DNA copies peaked at 1 d.p.i., decreased after 1 d.p.i., and then plateaued from 3 d.p.i. to 6 d.p.i. NP1 protein continued to increase in MA104 cells from 1 to 6 d.p.i. (Fig. 3E). Collectively, these results indicated that the complete life cycle of HBoV1 occurred in MA104 cells.

To analyze the susceptibility of MA104 cells to HBoV clinical strains, nine pharyngeal swabs from HBoV-infected patients were used to infect MA104 cells. qPCR detection of intracellular HBoV1 DNA showed that 5 of the 9 samples with high viral loads (Table S2) successfully infected the MA104 cells (Fig. 3F). These results suggested that MA104 is a permissive cell line for HBoV1 infection.

We attempted to treat HBoV1 infection in MA104 cells with cidofovir, which had been reported as an effective inhibitor for regulating parvovirus B19 replication (27, 28). The effect of cidofovir on HBoV1 replication was detected as a dose-dependent reduction in the amount of viral DNA at 72 h.p.i. (Fig. 3G), without causing cell cytotoxicity at concentrations up to 400 µM (Fig. 3H). Treatment with 400 µM cidofovir resulted in approximately 50% inhibition of HBoV1 replication (Fig. 3G). These results indicated that cidofovir had some inhibitory effect on HBoV1 and suggested that MA104 cell line can serve as a cell evaluation model for screening antiviral drugs against HBoV1.

### MA104 cell response to viral infection

**Fig 4:**
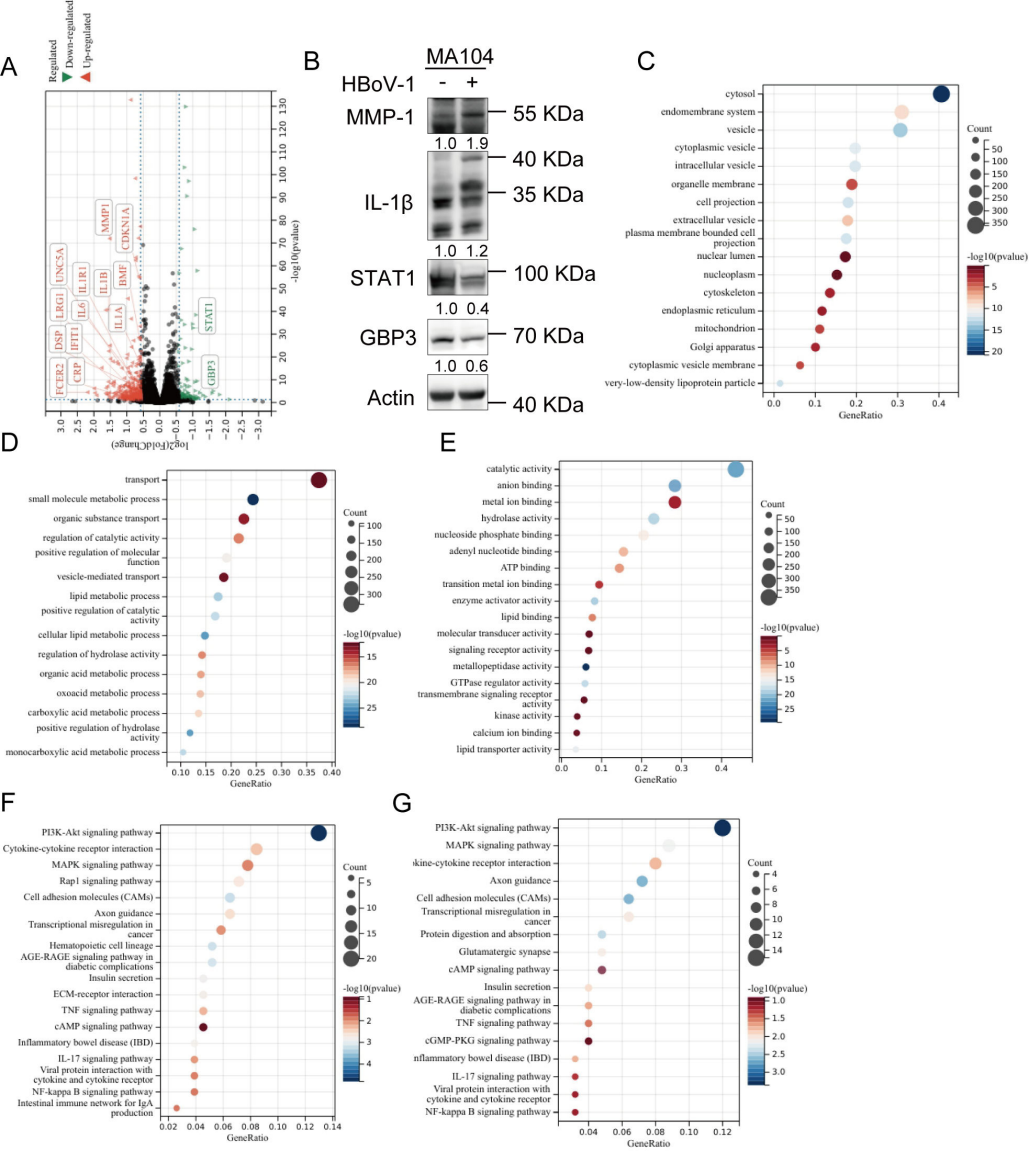
MA104 cell response to viral infection. (**A**) Volcano plot of differentially expressed genes (DEGs) in HBoV1-infected vs uninfected MA104 cell lysates (3,000 vge/cell for 96 h). The highlighted points indicate DEGs with both statistical significance (−log10(*P*-value), *x*-axis) and a large fold change (log2(fold change), *y*-axis). The dashed horizontal line indicates the *P* < 0.05 cutoff. The two dashed vertical lines indicate the fold change >1.5 cutoff for downregulated (green region) and upregulated (red region) genes. (**B**) Protein analysis of upregulated or downregulated genes in MA104 cells with HBoV1 infection by Western blotting. (**C–E**) Enriched GO. (**C**) cellular component (CC), (**D**) biological process (BP), and (**E**) molecular function (MF) terms associated with DEGs. (**F and G**) Enriched KEGG pathways of (**F**) DEGs or (**G**) upregulated genes.

To further confirm the HBoV1 infection in MA104 cells and better understand the interaction between the virus and host, the transcriptome of HBoV1-infected vs uninfected MA104 cells at 96 h.p.i. was compared using RNA-seq. HBoV1 infection upregulated 330 genes and downregulated 124 genes [fold change (FC) >1.5 and *P* < 0.05], as shown in the volcano plot (Fig. 4A). IFN-stimulated genes (ISGs), including CRP, IFIT1, LRG1, SLC1A1, and CDKN1A, were upregulated during HBoV1 infection (Fig. 4A), suggesting that viral infection elicited an antiviral response from the host cell. Genes associated with the inflammatory cytokines IL-2, IL-4, and IL-10, such as IL-1B, JAK3, IL1A, IL-6, CDKN1A, PTGS2, and MMP1, were also upregulated (Fig. 4A). The upregulated genes were also related to cytokine production (including IL-10 and IL-1β) and type II immune response, consistent with a previous study (2). Additionally, the expression of up-regulated genes such as IL-1β and MMP1 or down-regulated genes such as STAT1 and GBP3 protein level expression was confirmed by Western blotting assay, which was consistent with those of transcription level (Fig. 4B). These results suggested that HBoV1 infection activates host antiviral responses and induces inflammation.

Gene Ontology (GO) analysis of the differentially expressed genes (DEGs) was conducted. Regarding cellular components (CCs), cytosol, endomembrane system, cytoplasmic and intracellular vesicle, and nucleoplasm-related genes were enriched in the HBoV1 infection group, indicating that many membrane-related components and the vesicle system were involved in HBoV1 infection (Fig. 4C). In the category of biological processes (BPs), transport- and metabolism-related terms were enriched (Fig. 4D); the related genes might have a role in viral replication and virion maturation, as parvovirus vesicular transport through the endoplasmic reticulum and Golgi complex significantly accelerates cytolysis and progeny virion release (29). GO analysis of molecular functions (MFs) showed that catalytic activity, including hydrolase activity, enzyme activator activity, and metallopeptidase activity, may play a role in regulating HBoV1 infection at the transcript level (Fig. 4E).

KEGG analyses of DEGs or upregulated genes in HBoV1-infected MA104 cells both revealed that common targets were mainly enriched in the PI3K-Akt signaling pathway, cytokine–receptor interactions, and the MAPK signaling pathway (Fig. 4F and G), which are closely related to the regulation of viral infections, including DNA and RNA viruses and retroviruses (30, 31).

Collectively, these results demonstrated that membrane- and transport-related genes, as well as the PI3K-Akt and MAPK signaling pathways and immune responses, are modulated by HBoV1 infection.

### Suppression of IFN signaling enhances HBoV1 replication in MA104 cells

**Fig 5:**
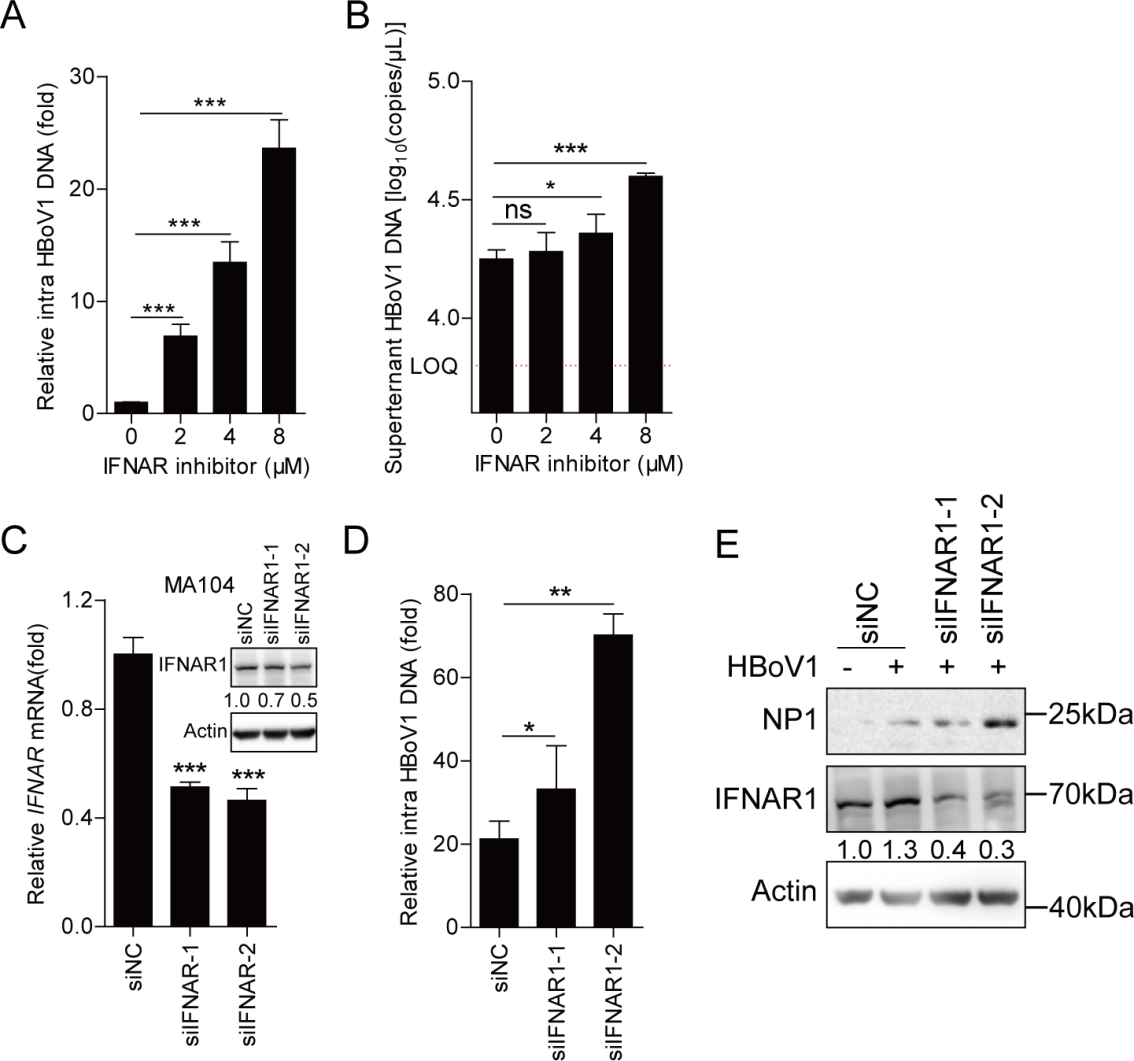
Suppression of IFN signaling enhances replication in MA104 cells. (**A and B**) MA104 cells were treated with 2, 4, or 8 µM IFN alpha-IFNAR-IN-1 (#HY12836) for 4 h and infected with HBoV1 for 96 h, and then (**A**) intracellular and (**B**) extracellular viral DNA copies were quantified. (**C**) MA104 cells were transfected with siIFNAR1 (siIFNAR1-1 or siIFNAR1-2) or siNC control, and IFNAR1 knockdown efficiency was evaluated by qRT-PCR and Western Blotting without HBoV1 infection. (**D and E**) MA104 cells were transfected with siIFNAR1 or siNC for 48 h, infected with HBoV1 for 96 h, and then (**D**) intracellular viral DNA copies and (**E**) NP1/IFNAR1 protein were detected. Data are representative of three independent experiments. Graphs show mean ± SD; *n =* 3. *P < 0.05; **P < 0.01; ***P < 0.001 (Student’s *t*-test).

As RNA-seq revealed that host antiviral immunity might be activated during HBoV1 infection, whether suppression of IFN signaling increased HBoV1 replication was assessed. First, IFN alpha-IFNAR-IN-1 inhibitor (#HY12836) dose-dependently enhanced HBoV1 replication in MA104 cells based on quantification of intracellular and extracellular viral DNA copies by qPCR (Fig. 5A and B). Second, two small interfering RNAs (siRNAs) specifically targeting IFNAR (siIFNAR1-1 and siIFNAR1-2), which were confirmed to knockdown IFNAR by RT-PCR and Western blotting (Fig. 5C), also enhanced HBoV1 replication in MA104 cells (Fig. 5D and E). These results suggested that suppression of IFN signaling improves HBoV1 replication.

## DISCUSSION

HBoV1 is an emerging pathogen, with an increasing number of cases reported worldwide. So far, here is a lack of a cell line suitable for HBoV1 infection. In this study, we found that MA104 cells are permissive to HBoV1 infection, and they supported the complete life cycle of HBoV1. Suppression of IFN signaling promoted viral genome replication in MA104 cells. Additionally, RNA-seq showed that host membrane-, vesicle-, and transport-related genes, as well as the PI3K-Akt and MAPK signaling pathways and immune responses have essential roles in regulating HBoV1 infection.

HBoV1 infects differentiated, polarized, primary HAE cells and tends to replicate in ALI cultures at both the apical and basal surfaces (2), indicating that the receptor for the virus is expressed on both ciliated apical and basal cells (9). Whereas, no HBoV1 infection of human lung cell lines has been reported. The cell lines (Caco-2 and HEK293T) that have been reported are all partially permissive for HBoV1 infection (22–24). Caco-2 (colon epithelial) cells support HBoV1 entry, but infectious progeny virions are not produced (25). In our study, NP1 was detected in very few cells (only MA104 [kidney epithelial] and RD [muscle rhabdomyosarcoma] cells), but not Caco-2 cells, further indicating that Caco-2 cells could only support partially infection life cycle of the virus. Based on NS1 mRNA and NP1 protein expression in RD cells (Table S1; Fig. 1B), we suspect that RD cells may also support HBoV1 the invasion, which warrants further investigation. HEK293T (kidney epithelial) cells produced high titers of infectious progeny virions upon transfection, but did not support HBoV1 entry, most likely due to a lack of receptors for the virus (22). In this study, complete life cycle analysis showed that HBoV1 could invade MA104 cells, replication, and produce infectious progeny virions. MA104 cells also supported the infection of HBoV clinical samples, demonstrating their wide range of applications. Moreover, the application for antiviral drug evaluation successfully provided a potential effective drug cidofovir to inhibit HBoV1 replication, which was worthy further investigation. Based on our preliminary data, we also found that HBoV1 can infect human and non-human primate cells, but not cells from other animals such as mice, reflecting its high host specificity.

In general, parvoviruses enter cells by receptor-mediated endocytosis, which involves a virion binding to specific receptors on the cell surface, followed by internalization of the virus into the host cell (2). The successful binding and internalization of HBoV1 in MA104 cells might reflect the fact that the parvovirus HBoV1 may utilize this pathway to enter cells (with an unknown receptor). Intracellular viral DNA copies significantly increased over 24 h.p.i. and then remained at a certain level. The replication kinetics are similar to those in Caco-2 cells (25), but different to those in primary HAE cells, which involve a delay in the initiation of HBoV1 replication (25, 32). It is worthwhile to study why the intracellular and extracellular viral DNA copies peaked at 24 h.p.i. after MA104 infection, while the viral NP1 protein was still low. Viral genome sequencing analysis (data not shown) showed that HBoV1 replication in MA104 cells did not result in significant mutations, which do occur in Caco-2 cells (25).

Upon viral infection, the cells have a complex response. The activation of the innate immune system is the first barrier for cells to fight viral infections. RNA-seq indicated that ISGs and inflammation-associated genes were upregulated, which might be induced by VP2 (33). This may be one of the reasons for the low replication level in MA104 cells. Suppression of IFN signaling using a small-molecular inhibitor or siRNAs was, indeed, effective in increasing viral replication in MA104 cells. Upregulated genes associated with the inflammatory cytokines IL-2, IL-4, and IL-10 might provide clues to the mechanism of lung injury caused by HBoV1 infection. HBoV1 infection increases the concentrations of IL-2, IL-4, IL-10, and IL-13 in patients (34, 35). The host membrane- and vesicle-related gene expression enriched during HBoV1 infection implies that the cell membrane system may be rearranged to favor viral replication. The cell membrane system has a role in parvovirus infection; for example, BV19 virions can be coated with phospholipid membrane to evade host immune responses and enhance membrane fusion-mediated transmission, and adeno-associated virus (AAV) virions require extracellular vesicles in order to be released from cells (36–38). Thus, in addition to the virology of HBoV1, the HBoV1-MA104 infection system could be used to study virus–cell interactions.

In conclusion, our study identified a HBoV1-permissive cell line, providing a viable cell model for research on HBoV1 infection, including identifying pathogenic mechanisms and antiviral drugs. The analysis of HBoV1–host interactions improves our understanding of the intracellular pathways involved in HBoV1 infection, which may be useful for future research on HBoV1 virus-host interaction.

## MATERIALS AND METHODS

### Reagents and antibodies

The small-molecule inhibitor IFN alpha-IFNAR-IN-1 (#HY12836) and cidofovir (#HY-17438) were purchased from MCE. Hoechst (#H1398) and Benzonase Nuclease (#88701) were sourced from Invitrogen. β-Actin monoclonal antibody (#66009-1), GAPDH monoclonal antibody (#60004-1-Ig), GBP3 recombinant antibody (#83534-1-RR), IL-1 beta polyclonal antibody (#26048-1-AP), STAT1 polyclonal antibody (#10144-2-AP), and IFNAR1 polyclonal antibody (#13083-1-AP) were obtained from Proteintech. MMP1 recombinant antibody (#ab134184) was purchased from Abcam. The antibodies against NS1 and NP1 (for immunofluorescence and Western blotting, respectively) were custom-produced by Sinobiological. The antibodies against NS1 and NP1 (for immunofluorescence and Western blotting, respectively) were custom-produced by Sinobiological. Two antigenic peptides of NP1 (Ac-YKRKGSPERGERKRHWC-NH2 and Ac-TRESTSGKKDNRTNC-NH2) were designed and *in vitro* synthesized. Two antigenic peptides of NS1 (Ac-CSVSTTYKPNKKKE-NH2 and Ac-CSADNSMYTDRASETS-NH2) were designed and *in vitro* synthesized. The rabbit polyclonal antibodies to the peptides were obtained by protein A and antigen affinity purification.

### Cell culture

HEK293T (ATCC #CRL-3216), MA104 (ATCC #CRL-2378.1), Caco-2 (ATCC #HTB-37), A549 (ATCC #CCL-185), HeLa (ATCC #CCL-2), and RD (ATCC #CCL-136) cells (American Type Culture Collection) were cultured in Dulbecco’s modified Eagle’s medium (DMEM) (Gibco) supplemented with 2 mM HEPES (Gibco), 10% fetal bovine serum (FBS) (Invitrogen), and 1% penicillin–streptomycin (Life Technologies). A172 cells (Procell #CL-0012) were cultured in DMEM. HT-29 cells (Shanghai Zhong Qiao Xin Zhou Biotechnology Co., Ltd. #ZQ0057) were cultured in RPMI-1640 (Gibco) supplemented with 10% FBS and 1% penicillin–streptomycin. All cells were cultured at 37°C in a humidified atmosphere with 5% CO_2_.

### Plasmids

The recombinant infectious clone pHBoV1-WH (39, 40) was a kind gift from Dr. H. Z. Wang and Dr. W. X. Guan (Wuhan Institute of Virology).

### Transfection for HBoV1 production and purification of intracellular and extracellular virions

HEK293T or MA104 cells seeded on 200 mm plates were transfected with pHBoV1-WH (50 µg) using Polyethylenimine Max transfection reagent (Mw 40,000) (Polysciences) for 72 h.

To purify the intracellular HBoV1 virions, the cells were collected with phosphate-buffered saline (PBS; pH 7.4), lysed by three rounds of freezing (−196°C) and thawing (37°C), and treated with Benzonase Nuclease (50 U/mL) for 1.5 h at 37°C. The cell lysate was then spun at 10,000*g* and 4°C for 30 min. The virions in the supernatant were further purified by using a 20% (wt/vol) sucrose cushion in PBS and ultracentrifugation at 100,000 × *g* and 4°C for 2 h. The pellet was resuspended in PBS.

To purify the extracellular HBoV1 virions, MA104 cell culture supernatant was digested using Benzonase Nuclease at 37°C for 1.5 h, centrifuged at 500 × *g* and 4°C for 10 min to remove the debris, precipitated using 8% (wt/vol) polyethylene glycol 8000, incubated overnight at 4°C, and centrifuged at 10,000 × *g* and 4°C for 1 h. The pellet was loaded onto a 20% (wt/vol) sucrose cushion in TNE buffer (10 mmol/L Tris-HCl, pH 8.0, 120 mmol/L NaCl, and 1 mmol/L EDTA) and ultracentrifuged at 100,000 × *g* and 4°C for 2 h. The pellet was then resuspended in TNE buffer.

Viral DNA was extracted using a TIANamp Virus DNA/RNA Fast Kit (TIANGEN), and the viral DNA copies were quantified by qPCR.

### Negative-stain electron microscopy

The intracellular or extracellular HBoV1 virions were purified as described in an earlier section. For transmission electron microscopy, 10 µL of the intracellular or extracellular virions was placed on a Formvar/Carbon film-coated grid and incubated for 1 min, and excess sample was then removed by touching the edge of the grid to a filter paper. Next, the grids were negatively stained using 2% phosphotungstic acid (pH 7.0) for 40 s. Excess liquid was absorbed with filter paper, and then the grids were dried naturally. The intracellular or extracellular virions were observed using a transmission electron microscope (Tecnai G2 Spirit) at an acceleration voltage of 120 kV.

### Quantitative polymerase chain reaction

Viral genomes were extracted from intracellular and extracellular (cell culture supernatant) virions using a TIANamp Virus DNA/RNA Fast Kit (TIANGEN Biotech) according to the manufacturer’s instructions. The viral DNA copies were quantified by qPCR using a Taq Pro HS Universal Probe Master Mix (Vazyme). Absolute quantification was conducted using a standard curve. The primer sequences are provided in Table S3.

### HBoV1 infection

Cells in medium containing 2% FBS and 0.5% dimethyl sulfoxide (DMSO) were incubated for 12 h with purified HBoV1 (3,000 vge/cell) and then washed three times with PBS. The medium was replaced with fresh medium containing 2% FBS and 0.5% DMSO. The cells and culture supernatants were collected at the indicated time points after three times washing with PBS.

### Evaluation of antiviral activity

MA104 cells (2 × 10^5^) in 12-well plates were treated with H_2_O or Cidofovir (400 µM, 200 µM, 100 µM, 50 µM) for 6 h before infection and then incubated with HBoV1 for 12 h at 37°C. After the absorption period, cells were washed with PBS for three times and cultured in fresh medium with various concentration of Cidofovir or not. At 96 h.p.i., the intracellular HBoV1 DNA level was detected.

### Quantitative reverse‐transcription polymerase chain reaction

Total RNA was extracted from cells using TRIzol reagent (Invitrogen) according to the manufacturer’s instructions to quantify HBoV1 NS1 mRNA and evaluate the IFNAR1 knockdown efficiency. One-step real-time qRT-PCR was conducted using a HiScript II One Step qRT-PCR SYBR Green Kit (Vazyme). β-Actin was used for normalization. Relative expression was calculated using the 2^−ΔΔ*Ct*^ method. The primer sequences are provided in Table S3.

### Western blotting

Western blotting was performed as previously described (41). Four antibodies against the HBoV1 NP1 protein were generated. Western blotting indicated that αNP1-3 and αNP1-4 antibodies specifically recognized the NP1 protein, and the latter antibodies were used for the following Western blotting assays (Fig. S3). Briefly, cells were lysed in lysis buffer for 30 min at 4°C and centrifuged at 12,000 *g*, 4°C for 10 min. The proteins were quantified, separated by 12% SD S-PAGE, electro-transferred to nitrocellulose filter membranes (Millipore), blocked for 1 h with 5% nonfat milk, incubated with antibodies, and visualized using horseradish peroxidase (HRP)-conjugated secondary antibodies (Jackson ImmunoResearch) and WesternBright Sirius HRP substrate (Advansta).

### Southern blotting

HBoV1 DNA replicative forms were detected by Southern blotting as previously described (42). Cells were collected, washed twice with PBS, lysed with TE buffer (10:10) (10 mM Tris-HCl, pH 7.5, and 10 mM EDTA) and 10% SDS for 30 min, subjected to Hirt DNA extraction via phenol–chloroform extraction, and either digested or not using DpnI restriction endonuclease. The DNAs were subjected to 1.1% agarose gel electrophoresis, and Southern blotting was then performed using HBoV1 dsDNA genome as the probe.

### Virus binding and internalization assays

HEK293T or MA104 cells (1 × 10^5^) were seeded on 24-well plates for 16 h. For the binding assay, the cells were washed twice with PBS and incubated on ice for 1  h with HBoV1 (20,000 vge/cell) in cold DMEM supplemented with 2% FBS. The cell culture supernatant was then removed, the cells were washed five times with cold PBS, lysed, and subjected to intracellular DNA extraction, and the viral DNA copies were then quantified by qPCR. For the internalization assay, the cells were washed five times and incubated at 37°C for 6 h with fresh medium containing 2% FBS; then, cells were chilled on ice and treated with 500 ng/mL proteinase K on ice for 1 h. After five additional washes with cold PBS, lysed, and subjected to intracellular DNA extraction, and the viral DNA copies were then quantified by qPCR.

### Immunofluorescence-based focus-forming assay

MA104 cells (1.5 × 10^4^) were seeded in 96-well plates for 16  h, the cells were infected with HBoV1 or fourfold gradient dilution virus, or the supernatants of the HBoV1 infected intracellular lysis by centrifugation × 350 *g* at 30°C for 30 min. At 96 h.p.i., cells were fixed in 4% PFA and then permeabilized with 0.3% Triton X-100. HBoV1 was probed using anti-HBoV1 NS1 antibody diluted 1:50 in PBS and incubated at 4°C overnight. Samples were washed with PBS and incubated with Alexa Fluor 561-conjugated secondary antibodies (Invitrogen), which were added for 1 h at 37°C. After washing and counterstaining with DAPI (Invitrogen), the fluorescence of sample was observed with OperettaCLS (PerkinElmer). The virus titer is calculated according to the following formula: virus titer (FFU/mL) = ((average number of fluorescent foci per well × dilution degree)/volume).

### RNA-mediated interference

To knockdown IFNAR1, MA104 cells (4 × 10^5^) cultured in 6-well plates were transfected for 24 h with 20 nM of siIFNAR1-1 (5′-CGCUCAAGCUGAACAUUUATT-3′), siIFNAR1-2 (5’- GGAUUAUCCACUGAUUUAUTT-3′), or scrambled (non-silencing) siRNA (negative control) using Lipofectamine RNAiMAX reagent (Invitrogen) according to the manufacturer’s instructions. To keep IFNAR silencing efficiency for the duration of the test, the cells were split at 24 h post-transfection and transfected with the same siRNA again.

### Cell viability assay

MA104 cells (1.5 × 10^4^) were seeded in 96-well plates for 16  h. Compounds in a twofold dilution series were added to the cells, and four wells were performed in parallel. After 72  h, 100 µL of CellTiter-Glo reagent (Promega) was added to each well and subjected to shaking for 5 min, then the luciferase activity was evaluated.

### RNA-seq

HBoV1-infected (3,000 vge/cell) and uninfected MA104 cells were harvested at 96 h.p.i. Total RNA was extracted with TRIzol reagent. RNA-seq was performed by Beijing Qingke Biotechnology Co., Ltd. Briefly, sequencing libraries were generated for sequencing on an Illumina HiSeq 4000 platform, yielding 150 bp paired-end reads.

### Statistical analyses

Statistical analyses were performed using GraphPad Prism. Pairs of groups were compared using two-tailed Student’s *t*-tests. For multiple group comparisons, a one-way analysis of variance (ANOVA) was conducted. Data are presented as mean ± SD. The differences were considered to be statistically significant at *P < 0.05, **P < 0.01, ***P < 0.001.

## Data Availability

The RNA-seq raw data have been uploaded in NCBI SRA. The BioProject ID is PRJNA1136809, and the BioSample IDs for samples with or without HBoV1 infection are SAMN42554197, SAMN42554198, SAMN42554199, SAMN42554200, SAMN42554201, and SAMN42554202.

## SUPPLEMENTAL MATERIAL

Supplemental figures jvi.01539-24-s0001.pdf Figures S1 to S3.

Supplemental tables jvi.01539-24-s0002.pdf Tables S1 to S3.

ASM does not own the copyrights to Supplemental Material that may be linked to, or accessed through, an article. The authors have granted ASM a non-exclusive, world-wide license to publish the Supplemental Material files. Please contact the corresponding author directly for reuse.
