# Supplementary material for: MA104 cell line is permissive for human bocavirus 1 infection
Source: J Virol. 2025 Jan 23;99(2):e01539-24. doi: 10.1128/jvi.01539-24 (PMC11852709; doi:10.1128/jvi.01539-24)
Supplement: Supplemental figures — Figures S1 to S3. [file jvi.01539-24-s0001.pdf]

Supplementary figure legend

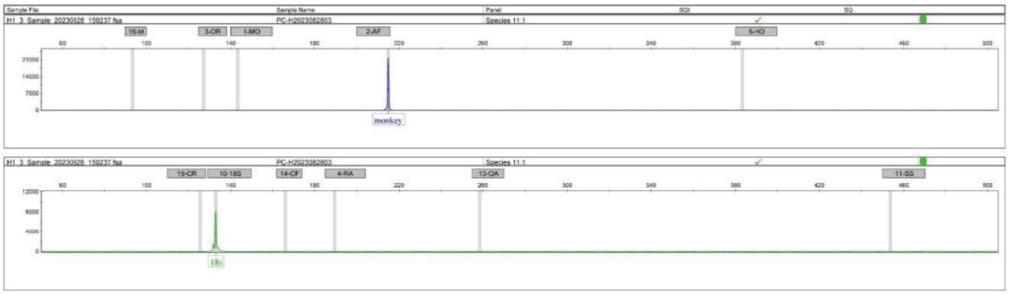

Figure S1 Confirmation of MA104 cells' species. DNA from MA104 cells was extracted. Mitochondrial cytochrome c oxidase subunit I (COI) gene (and internal reference gene 18S rRNA) was amplified using a combination of fluorescent primers for various species (including the monkey-specific primer 2-AF). The amplified products were detected and analyzed by capillary electrophoresis using GenReader 7010.

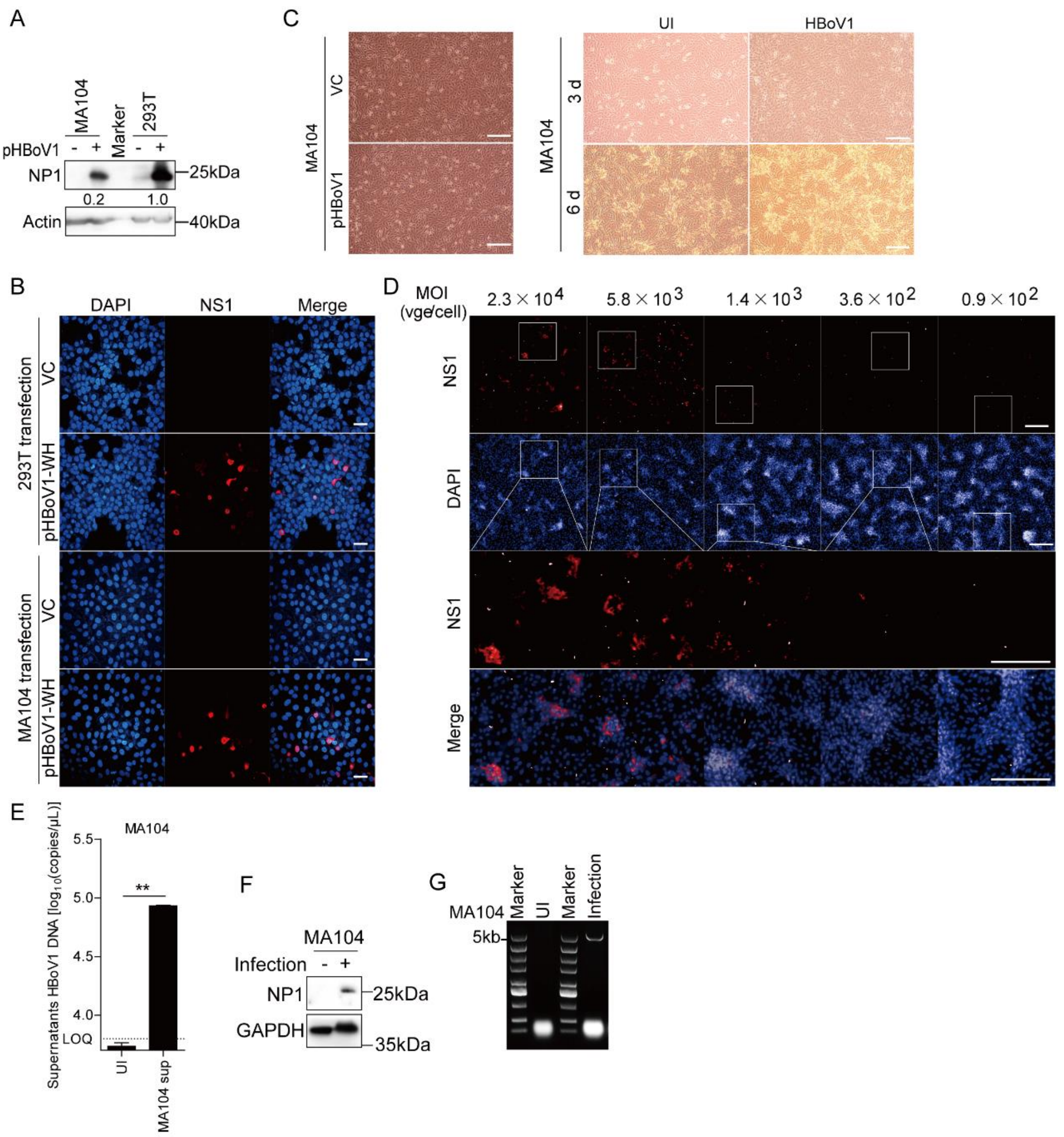

Figure S2 HBoV1 infectious characterization in MA104 cells. (A) HEK293T or MA014 cells were transfected with pHBoV1-WH for 48 h, and then NP1 protein was analyzed. **(B)** HEK293T or MA104 cells were transfected with pHBoV1-WH or VC for 48 h, probed with the DNA-binding dye DAPI (blue) and anti-NS1 antibody (red) in an immunofluorescence analysis, and then subjected to confocal microscopy. Scale bar, 10 µm. **(C)** Observation of cell status of MA104 cell along with pHBoV1 transfection at 48 h or HBoV1 infection at 3 d.p.i and 6 d.p.i. Microscope was taken at 10× magnification. Scale bar, 100 µm. **(D)** MA104 cells were incubated with 50 uL HBoV1 (Copies=1.85×10^{10} vge/mL, copies number calculation based on HBoV1 standard curve) or 4-fold

gradient dilution. After 96 h.p.i, the virus titer was performed by fluorescent focus assay, probed with the DNA-binding dye DAPI (blue), anti-NS1 (red). Fluorescence microscopy images involve 10× magnification. Scale bar, 100 μm. Virus titer (FFU/mL) = (average number of fluorescent foci per well × dilution degree)/volume, ((60×256)/0.1 mL=1.53×10^5 FFU/mL). **(E&F)** MA104 cells were reinfected or not using HBoV1-infected MA104 cell supernatant (concentrated 100×), incubated for 8 days post infection, and then **(E)** extracellular viral DNA copies and **(F)** NP1 protein were detected. **(G)** MA104 cells were infected or not with HBoV1 (3000 vge/cell) for 12 h, and viral genome PCR amplicons were subjected to gel electrophoresis at 96 h.p.i. Graphs show mean ± SD; ** *P<0.01;* (Student's *t*-test).

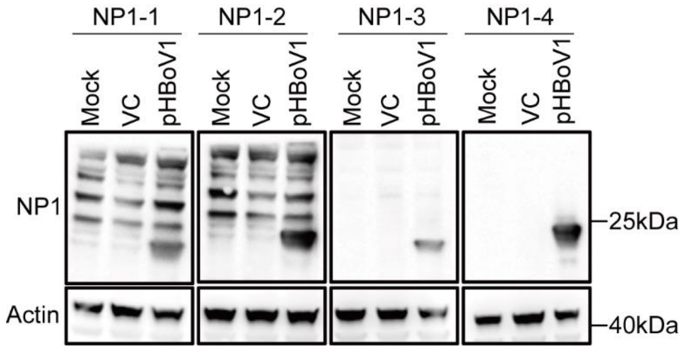

Figure S3 Specificity of four antibodies against HBoV1 NP1 protein. Western blotting of HEK293T cells transiently transfected for 48 h with pHBoV1-WH or empty vector control (VC) (1 μg each). Data are representative of three independent experiments.
