# Supplementary material for: MA104 cell line is permissive for human bocavirus 1 infection
Source: J Virol. 2025 Jan 23;99(2):e01539-24. doi: 10.1128/jvi.01539-24 (PMC11852709; doi:10.1128/jvi.01539-24)
Supplement: Supplemental tables — Tables S1 to S3. [file jvi.01539-24-s0002.pdf]

Table S1. Cell lines tested for HBoV1 infection susceptibility

Number	Cell line	Cell type	Tissue	HBoV1 NS1 mRNA (Ct value)	HBoV1 NS1 mRNA
	Human				
1	HEK-293	Epithelial	Kidney	31.8	-
2	HEK293T	Epithelial	Kidney	32.5	-
3	SW-13	Epithelial	Kidney	29.5	±
4	A549	Epithelial	Lung	27.9	±
5	BEAS-2B	Epithelial	Trachea	30.9	±
6	NCI-H292	Epithelial	Lung	32.4	-
7	Calu-3	Epithelial	Lung	29.3	±
8	HEp-2	Epithelial		31.3	±
9	HeLa	Epithelial	Cervix	28.4	±
10	A172	Fibroblast	Brain	28.6	±
11	U251	Fibroblast	Brain	31.7	-
12	U-87 MG	Epithelial	Brain	30.1	±
13	SH-SY5Y	Epithelial	Bone marrow	28.9	±
14	T98G	Fibroblast	Brain	32.7	-
15	RD	Rhabdomyosarcoma	Muscle	28.3	±
16	Raji	B lymphocyte	Lymphoblast	32.9	-
17	**Caco-2**	**Epithelial**	**Colon**	**26.9**	**Positive control**
18	HCT-8	Epithelial	Colon	31.0	±
19	HCT-15	Epithelial	Colorectum	31.7	-
20	**HT-29**	**Epithelial**	**Colorectum**	**26.0**	**+**
21	RKO	Epithelial	Colorectum	31.1	±
22	Hutu-80	Epithelial	Dodecadactylon	33.3	-
23	SW480	Epithelial	Rectum	30.7	±
24	SW579	Epithelial	Thyroid	31.2	±
25	MCF7	Epithelial	Breast	30.5	±
26	MDA-MB-231	Epithelial	Breast	29.7	±
27	AC16	Fibroblast	Heart	32.1	-
28	Huh-7	Epithelial	Liver	29.4	±
29	Huh-7.5.1	Epithelial	Liver	28.6	±
	Mouse				
30	CT26.WT	Fibroblast	Colon	31.8	-
	Monkey				
31	LLC-MK2	Epithelial	Kidney	33.6	-
32	**MA104**	**Epithelial**	**Kidney**	**25.0**	**+**

33	Vero E6	Epithelial	Kidney	31.3	±
	Dog				
34	MDCK	Epithelial	Kidney	33.3	-
	Pig				
35	PK-15	Epithelial	Kidney	32.3	-
	Cow				
36	EBTr(NBL-4)	Fibroblast	Trachea	31.3	-

Caco-2 cell line served as positive control for HBoV1 infection. -, +, and ± indicate no transcription (Ct value > limit of quantitation, i.e., 31.6), higher transcription (Ct value < Caco-2 cells' value), and lower transcription (Ct value > Caco-2 cells' value), respectively, after HBoV1 infection.

Table S2. Patient information

**Patient**	**Gender**	**Age**	**Sample type**	**HBoV1 copy number/(100uL)**
1	Male	2	Throat swab	1.1×10^{5}
2	Male	2	Throat swab	6.9×10^{4}
3	Male	3	Throat swab	3.6×10^{4}
4	Male	3	Throat swab	4.6×10^{4}
5	Male	4	Throat swab	1.1×10^{6}
6	Male	5	Throat swab	0.9×10^{4}
7	Female	1	Throat swab	7.1×10^{4}
8	Female	4	Throat swab	3.9×10^{5}
9	Female	5	Throat swab	3.1×10^{4}

Table S3. Primer sequences

**Gene name**	**Primer**	**Sequences (5'-3')**
NS1	F	GCACAGCCACGTGACGAA
	R	TGGACTCCCTTTTCTTTTGTAGGA
	Probe	FAM-TGAGCTCAGGGAATATGAAAGACAAGCATCG-BHQ
IFNAR1	F	GACCCTAGTGCTCGTCACCTTG
	R	AGATTCATCACTCCTGTTCCACCTC
Actin	F	GTGACGTTGACATCCGTAAAGA
	R	GCCGGACTCATCGTACTCC

F: forward primer; R: reverse primer.
